# Gambling and self-reported changes in gambling during COVID-19 in web survey respondents in Denmark

**DOI:** 10.1016/j.heliyon.2021.e07506

**Published:** 2021-07-08

**Authors:** Anders Håkansson

**Affiliations:** a Lund University, Faculty of Medicine, Department of Clinical Sciences Lund, Psychiatry, Lund, Sweden; b Region Skåne, Malmö Addiction Center, Clinical Gambling Disorder Unit, Malmö, Sweden

**Keywords:** Gambling disorder, Problem gambling, Covid-19, Behavioral addiction, Mental health, Pandemic

## Abstract

Problem gambling is among the public health hazards which may increase due to the COVID-19 pandemic and its consequences on society. Results from a few countries have hitherto been diverse with respect to gambling during the pandemic. The present study aimed to study gambling behaviors during COVID-19 in Denmark, with the same methodology as previously used in Sweden, and also to provide a comparison to previously published Swedish data. A web survey was answered by 2,012 individuals, from December 2, to December 10, 2020. Four percent reported increased gambling habits, and three percent reported decreased gambling, during COVID-19. Self-reported increase in gambling was associated with spending more time at home, mental distress, and higher gambling severity. Prevalence of self-reported, increased gaming during COVID-19 was significantly lower in Denmark than in Sweden. Although the overall changes in gambling patterns during COVID-19 may be limited, people who increase their gambling during the pandemic may be at particular risk. Stakeholders should pay attention to individuals who report altered gambling habits during the pandemic.

## 1 Introduction

The global pandemic spread of the SARS-CoV-2 virus, causing the COVID-19 disease, is suspected to have a substantial negative impact on mental health (Xiong et al., 2020). Thus, a number of public health hazards are suspected to worsen as a consequence of COVID-19, and researchers have expressed fear of increasing addictive behaviors (MacMillan et al., 2021). Problem gambling may be one of these public health consequences of the pandemic (Håkansson et al., 2020).

During the spring of 2020, it was suspected that gambling patterns in the population might change due to mental distress and changes in everyday habits, both as a consequence of the pandemic and related virus-preventing restrictions (Håkansson et al., 2020). In Sweden, a general population survey demonstrated that a small minority of respondents, four percent, reported increased gambling during the COVID-19 period, and that this group was characterized by a high degree of problem gambling (Håkansson, 2020a). A repeated study in Sweden, in November, 2020, displayed very similar results, with a somewhat larger group (six percent) self-reporting an increase in gambling, and with high rates of problem gambling in this group (Håkansson and Widinghoff, 2021). In the same setting, overall gambling data from the population indicated some shift in the gambling types used during the first months of the pandemic (Håkansson, 2020b). In Ontario, Canada, land-based casino gamblers shifted towards online gambling mainly if they had some degree of online gambling before (Price, 2020). In contrast, in Australia, a shift in gambling patterns during COVID-19 was not seen (Gainsbury et al., 2020). Despite the fear of possible increases in gambling, being a problem gambler during the pandemic may paradoxically even be relieving, if one's preferred gambling types are locked down (Donati et al., 2021). Altogether, trends in gambling during COVID-19 may be bidirectional; on one hand, home confinement and lock-down in land-based gambling venues may turn gamblers towards online chance-based games, whereas in contrast, the lock-down of sports and land-based venues may instead decrease gambling in many individuals who primarily prefer those gambling types (Håkansson et al., 2020).

Only a few countries world-wide have assessed gambling during COVID-19. Clearly, different countries have seen very diverse strategies against virus transmission. One example of two neighbor countries with markedly diverse strategies have been Denmark and Sweden, two countries with otherwise similar welfare systems. Denmark is among the countries addressing the pandemic with different phases of lock-down strategies, in contrast to Sweden, where formal lock-down procedures were never used (Yarmol-Matusiak et al., 2021). For example, in contrast to Denmark, Swedish restaurants, stores and shopping malls remained open throughout 2020 (Mens et al., 2021). In Denmark, after a first detected case in late February (Rohde et al., 2020), COVID-19 disease during the spring of 2020 reached a top impact in Denmark in early April (Clotworthy et al., 2020). The impact of COVID-19 again increased substantially during the autumn of 2020 and winter 2020/2021, with a new peak of virus transmission in mid-December, 2020. Likewise, the daily number of COVID-19-related deaths reached one peak in early April, 2020, and one new peak in mid-January, 2021 (Statens Serum Institut), with a death tool around twice the peak level of April. Due to the COVID-19 pandemic, formal lock-down in Denmark was announced on March 11, 2020 (Rohde et al., 2020). Early effects on mental distress in the general population was reported during lock-down measures, with more pronounced effects in women than in men (Sönderskov et al., 2020a). After partial re-opening of society in mid-April (including school openings from April 15), mental distress was reported to decrease again (Sönderskov et al., 2020b). Overall, only modest effects on mental health were reported during the spring phase of COVID-19 in Denmark, with effects mainly in the elderly, and on worry about the health of loved ones (Clotworthy et al., 2020). Due to an increase in virus transmission, new lockdown procedures were decided in specific regions from early December, 2020, and thereafter intensified in broader areas of the country during December, 2020, to at least February, 2021. Lockdown procedures in the country have involved the closing of stores other than for food and medications and similar, home schooling, travel restrictions, and bans against larger private gatherings.

Based on the mixed findings from studies carried out so far, uncertainty remains with respect to whether gambling habits may have changed as a consequence of the pandemic. In addition, it is of interest to compare the development of gambling across two settings with different types of restrictions during COVID-19. Therefore, the present study aimed to study self-reported changes in gambling behaviors, and correlates of such changes, in the Danish general population. Also, a secondary aim was to compare changes in gambling during COVID-19 in Denmark to the corresponding changes in the study from the neighbor country, Sweden, using similar methodology and inclusion criteria as here (Håkansson, 2020a; Håkansson and Widinghoff, 2020a, b).

## 2 Method

This study is a self-report web survey carried out in the general population in Denmark, addressing individuals enrolled in the web panel of the market research company Userneeds, located in Denmark. Based on Danish research ethics legislation, ethical approval is not required for anonymous survey studies which do not collect biological meterial. The questionnaire was distributed electronically by Userneeds to its web panel members, and the answers provided were sent to the companies Patient Information Broker AB and I-Mind Consulting AB, both located in Sweden, which collected study data without knowledge of the identity of respondents. A similar study of gambling data from the Danish population has been reported previously (Håkansson et al., 2019). Web panel members of Userneeds’ web panel regularly receive offers to participate in market surveys and similar polls. For the completion of a survey like the present one, the respondent receives credits to be used in an incentive system, corresponding to a value of around 1 Euro for the present survey.

In the communication with potential respondents, the survey was entitled ‘The COVID-19 pandemic and gambling habits’. Potential respondents were web panel members aged 18 years of older, and web panel members in each age group (see Table 1) were addressed until fairly representative quotas of age groups were reached. The study aimed to recruit respondents until about 2,000 complete responses had been obtained. In order to allow for comparison with the preceding studies from Sweden (Håkansson, 2020a; Håkansson and Widinghoff, 2020a, b), the distribution of age groups was aimed to be as similar as possible to these studies. The survey was carried out from December 2 to December 10, 2020. Duplicates, i.e. responses detected by the web panel provider as being sent from the same IP address, were handled in a way where the first response was included (if complete). Two complete and three partial registrations were therefore excluded because of being duplicates of a first complete answer, and along with 25 complete and four partial registrations which were duplicates of a first partial registration. The researcher was unaware of the IP addresses or geographical locations of respondents, and therefore no responses could be linked to any personal information.

**Table 1:** Sample characteristics, full sample (N = 2,012) in Denmark.

	% (n)
Gender	
- Male	54 (1,089)
- Female	46 (921)
- Wish not to report	0 (2)
Age groups (years)	
- 18–24	9 (181)
- 25–29	10 (202)
- 30–39	18 (355)
- 40–49	19 (385)
- 50–59	20 (400)
- 60+	24 (489)
Income (DKK)∗	
- -10,000	13 (257)
- 10,000–15,000	12 (251)
- 15,000–20,000	13 (263)
- 20,000–25,000	12 (233)
- 25,000–30,000	14 (278)
- 30,000–35,000	11 (229)
- 35,000–40,000	8 (152)
- 40,000–45,000	6 (118)
- 45,000–50,000	3 (61)
- 50,000+	8 (170)
Past-year gambling	
- Online casino	10 (202)
- Land-based casino	4 (84)
- Online horse betting	3 (64)
- Land-based horse betting	3 (56)
- Online sports betting	17 (338)
- Land-based sports betting	8 (164)
- Online poker	4 (80)
- Electronic gambling machines	6 (114)
- Online bingo	7 (131)
Psychological distress (Kessler-6 score >4)∗∗	
- Yes	42 (855)
- No	55 (1,116)
- Missing	2 (41)
Living conditions	
- with partner, no children	39 (784)
- with partner and children	25 (496)
- alone, no children	26 (528)
- alone with children	6 (113)
- with parents	5 (91)
Occupation	
- working	50 (1,014)
- studying	11 (225)
- unemployed	5 (99)
- temporarily unemployed due to COVID	1 (22)
- retired	26 (531)
- sick-leave	2 (50)
- other	4 (71)

∗ Local currently, 1 Danish Krona (1 DKK) corresponds to approximately 0.16 US Dollar (USD).

∗∗ Describing a level of at least moderate psychological distress, according to a score of five or more on Kessler-6, a six-item instrument describing symptoms of mental health (Furukawa et al., 2003).

A secondary analysis involved a direct comparison of the present dataset with the sample collected in Sweden, in November, 2020, and which was collected using the same methodology and inclusion criteria as in the present study. The comparison data has been published previously (Håkansson and Widinghoff, 2020a, b), and included a total of 2,029 individuals, among whom 1,301 were analyzed in the subset of gamblers (after excluding individuals who endorsed the option ‘do not gamble, neither now nor before COVID-19’. Thus, the present study involved also a direct statistical analysis with that previously published Swedish dataset. The Swedish study did not require ethical permission, based on the fact that is does not involve data which can be directly or indirectly linked to an identified individual.

### 2.1 Study variables

The following variables were included in the study:

• Gambling habits. Past-year history of any gambling on each of the following gambling types: online casino, land-based casino, online sports betting, land-based sports betting, online horse race betting, land-based horse race betting, electronic gambling machines, online poker, and online bingo.
• Problem gambling. This included the Problem Gambling Severity Index, nine-item scale used for the measure of problematic gambling patterns. From a score of 0–3 on each item, out of a total score of 0–27, a score of 8 and above is categorized as problem gambling, 3–7 moderate-risk gambling, 1–2 low-risk gambling, and 0 represents no risk (PGSI, Wynne and Ferris, 2001).
• Behavioral changes during COVID-19. A question was asked about whether the respondent's gambling had increased, decreased or remained unchanged during COVID-19 (or whether the individual did not gamble at all, neither before nor during the pandemic, here referred to as ‘non-gamblers’). In addition, the corresponding changes in alcohol consumption, and time spent at home, were asked. In addition, past-year participation in each type of gambling was reported.
• Mental distress was measured with a six-item scale, the Kessler-6, assessing anxiety and depression symptoms over the past six months. Items are scored 0–4, and from a total score of 0–24, scoring five or above is classified as moderate mental distress (Kessler-6, Furukawa et al., 2003). Missing values for any of the six items were seen in 71 participants. Among them, 30 could still be included, as their scores from available items reached above cut-off for mental distress (n = 27), or because they had one missing item and a total score of zero from the remaining five items (n = 3). Remaining 41 individuals were excluded from analyses involving mental distress. From the analysis of gender, the two individuals who answered ‘other/do not wish to answer’ were excluded in analyses involving gender.
• Treatment needs for addictive behaviors. Dichotomous questions were asked about whether the respondent had ever perceived a need to seek treatment for alcohol problems, drug problems, or problem gambling, respectively.
• Socio-demographic data included gender (male, female, ‘other/prefer not to answer’), occupation (working, studying, unemployed, temporarily unemployed due to COVID-19, retired, sick-leave, or other), living conditions (alone and/or with partner, with or without children, or with one's parents), and monthly income levels (in 5,000 DKK intervals, referring to the Danish currency (1 DKK corresponding to around 0.16 USD). Also, age was reported, and in order to facilitate statistical analyses and to fully maintain confidentiality, age was reported in discrete categories, starting from the legal gambling age of 18 (18–24, 25–29, 30–39, 40–49, 50–59, and 60 + years). In order to facilitate interpretation of analyses, and in order to avoid smaller response categories in the regression analyses, for data describing occupation, this was categorized into those in active occupation (working and studying) in comparison to all others, and living conditions were categorized into living alone (without partner or children) vs all others.

The same variables were used as in previous studies on gambling during COVID-19 in Sweden (except for the item describing a specific Swedish self-exclusion system addressed specifically in the Swedish studies, Håkansson, 2020a; Håkansson and Widinghoff, 2021).

### 2.2 Setting

The present study was carried out in Denmark, where problem gambling, as measured with a brief screening tool (the ‘Lie-Bet’), has been reported to be present in around one percent of the population (Ekholm et al., 2014), and generally, rates of problem gambling have been reported around one percent or slightly less. Denmark, similar to other Scandinavian nations, has relatively pronounced regulation of gambling, including a strong role of the Danish state-owned gambling operator (Spångberg and Svensson, 2020). In an international comparison, although prevalence data have been measured with diverse methods, problem gambling in Denmark has demonstrated figures in the lower range. Also, in Denmark, figures have typically been lower than in corresponding surveys from Sweden, where a total of around two percent problem gamblers has traditionally been reported (Calado and Griffiths, 2016), even though rates of problem gambling in Sweden may have decreased slightly in recent years (Abbott et al., 2018).

### 2.3 Statistical methods

Differences between the respondents reporting increased gambling were compared to groups of respondents reporting decreased or unchanged gambling, using chi-square tests for all included variables. Also, the same analyses were carried out when excluding individuals who reported to be non-gamblers both during and prior to the pandemic. Variables which were significantly (p < 0.05) different between gambling increasers and other respondents were entered in a logistic regression analysis, one for all included respondents, and one excluding non-gamblers. Results of logistic regression analyses were reported using odds ratios with 95 percent confidence intervals. Remaining data of problem gambling, rates of respondents reporting increase/decrease for each gambling type, and new initiated gambling types during COVID-19, was reported descriptively. In logistic regression, age was analyzed as a continuous variable describing increasing age groups, occupation was dichotomized into irregular occupation (non-working/studying) vs working/studying, psychological distress was dichotomized into moderate distress (Kessler-6 score of five or more) vs below moderate distress, gambling severity was analyzed as a continuous variable of increasing PGSI levels (no risk, low risk, moderate risk, and problem gambling), and questions about increased time spent at home and increased alcohol drinking were dichotomized into ‘increased’ vs all others (unchanged, decreased and others).

The comparison between the present dataset and the previously published Swedish data (Håkansson and Widinghoff, 2021) was made using chi-square analyses for a number of variables: gender, age group, proportion of active gamblers and proportion endorsing moderate risk or problem gambling, proportion reporting increased gambling, increased alcohol drinking and increased time at home during COVID-19, and the past-year prevalence of gambling for each of the gambling types assessed here. These comparisons were made both for the two entire samples, and for the two sub-samples of respondents who did not report being non-gamblers.

All calculations were carried out in the SPSS statistics software, version 25.0.

## 3 Results

Sample characteristics are reported in Table 1. From the 2,012 respondents included, 54 percent (n = 1,089) were male, and 46 percent (n = 921) female (two individuals reported other or did not wish to answer). A large majority of respondents reported spending either slightly more (39 percent, n = 785) or much more (41 percent, n = 828) time at home during COVID-19. Nine percent (n = 183) reported drinking more alcohol during COVID-19, 15 percent (n = 310) reported drinking less, 62 percent (n = 1,242) reported unchanged consumption, and 14 percent (n = 277) reported no alcohol consumption during or prior to the pandemic.

### 3.1 Changes in gambling during COVID-19 and correlates of increased gambling

Four percent (n = 76) reported increased gambling during COVID-19, three percent (n = 62) reported decreased gambling, 48 percent (n = 960) reported unchanged gambling, and 45 percent (n = 914) were non-gamblers. Eighty-five percent (n = 1,704) of respondents were categorized with no risk gambling, seven percent (n = 132) were low-risk gamblers, three percent (n = 65) were moderate-risk gamblers, and six percent (n = 111) were problem gamblers. Thus, altogether, nine percent (n = 176) were at least moderate-risk gamblers. Moderate-risk/problem gamblers represented 45 percent (n = 34) of those reporting an increase during COVID-19, 35 percent (n = 22) of those reporting a decrease, five percent (n = 50) of those reporting unchanged gambling during COVID-19, and one percent (n = 5) of those reporting to be non-gamblers.

The proportion of respondents who reported an increase and a decrease, respectively, was two and two percent for online casino, respectively, two and four percent for online sports betting, one and four percent for land-based sports betting, one and one percent for online horse betting, one and two percent for land-based horse betting, two and two percent for online lotteries, two and five percent for land-based lotteries, and one and four percent, respectively, for electronic gambling machines.

Respondents reporting increased gambling, compared to all other respondents, were more likely to report increased alcohol consumption (22 vs 9 percent, p < 0.001), to have higher gambling severity level (<0.001, chi-square linear-by-linear), spending more time at home (93 vs 80 percent, p < 0.01), to be younger (p < 0.001, chi-square linear-by-linear), to be in a non-regular occupation (18 vs 8 percent, p < 0.01), and to screen positive for mental distress (87 vs 42 percent, p < 0.001). However, those reporting an increase did not differ with respect to gender (47 vs 54 percent men, p = 0.22), living alone without children (22 vs 26 percent, p = 0.43), or monthly income (p = 0.49, chi-square linear-by-linear).

In the logistic regression (n = 1,971), increased gambling was independently associated with spending more time at home, higher gambling severity, and mental distress, whereas associations with increased alcohol consumption, occupation and age were no longer significant (Table 2).

**Table 2:** Logistic regression analyses of variables associated with the reporting of increased gambling, in all respondents with available data (n = 1,971) and in the sub-sample of gamblers (n = 1,098) in Denmark.

	All subjects with available data. OR (95 percent confidence interval), n = 1,971.	Subjects with available data, non-gamblers excluded. OR (95 percent confidence interval), n = 1,098.
More time at home	4.77 (1.78–12.80)	4.66 (1.74–12.46)
Gambling severity, higher	3.48 (2.76–4.39)	2.74 (2.11–3.56)
Mental distress	2.94 (1.34–6.44)	2.78 (1.25–6.15)
Increased alcohol	1.43 (0.72–.86)	1.55 (0.77–3.14)
Age groups	1.11 (0.92–1.35)	1.13 (0.90–1.41)
Irregular occupation	1.81 (0.87–3.75)	1.70 (0.82–3.54)
Male gender	-	0.58 (0.32–1.03)

When excluding non-gamblers (n = 1,098), reporting increased gambling was associated with younger age (p < 0.001, chi-square linear-by-linear), irregular occupation (18 vs 8 percent, p < 0.01), spending more time at home (93 vs 77 percent, p < 0.001), increased alcohol consumption (22 vs 8 percent, p < 0.001), higher gambling severity (p < 0.001, chi-square linear-by-linear), with mental distress (87 vs 41 percent, p < 0.001), and also with female gender (53 vs 33 percent women, p < 0.001), but not with income (p = 0.14, chi-square linear-by-linear), or living alone (22 vs 25 percent, p = 0.64).

In the logistic regression analysis in only gamblers (n = 1,075), increased gambling remained significantly associated with spending more time at home, higher gambling severity, and mental distress, whereas there was a nearly significant association with female gender (p = 0.06). No significant association with alcohol consumption, occupation and age were seen in this regression (Table 2).

For each of the gambling types assessed in the study, past-year gambling was more common in those reporting increased gambling during COVID-19, than in others; online casino (61 vs 8 percent, p < 0.001), land-based casino (20 vs 4 percent, p < 0.001), online horse betting (22 vs 2 percent, p < 0.001), land-based horse betting (16 vs 2 percent, p < 0.001), online sports betting (53 vs 15 percent, p < 0.001), land-based sports betting (28 vs 7 percent, p < 0.001), online poker (28 vs 3 percent, p < 0.001), electronic gambling machines (28 vs 5 percent, p < 0.001), and online bingo (32 vs 6 percent, p < 0.001).

### 3.2 New gambling patterns during COVID-19

The proportion of respondents who initiated new gambling types during COVID-19 were the following; online casino 3 percent (n = 62), land-based casino 2 percent (n = 34), online horse betting 2 percent (n = 32), land-based horse betting 1 percent (n = 28), online sports betting 3 percent (n = 54), land-based sports betting 1 percent (n = 29), online poker 1 percent (n = 28), electronic gambling machines 1 percent (n = 24), and online bingo 2 percent (n = 36).

The proportion of moderate-risk/problem gamblers among those initiating new gambling types during COVID-19 were 52 percent (n = 32) for online casino, 71 percent (n = 24) for land-based casino, 69 percent (n = 22) for online horse betting, 75 percent (n = 21) for land-based horse betting, 50 percent (n = 27) for online sports betting, 55 percent (n = 16) for land-based sports betting, 71 percent (n = 20) for online poker, 71 percent (n = 17) for electronic gambling machines, and 33 percent (n = 12) for online bingo.

### 3.3 Denmark vs Sweden comparison

The two samples did not differ with respect to age distribution (p = 0.50), whereas the Danish respondents were more likely to be male (54 vs 48 percent, p < 0.001). Swedish respondents were more likely to be gamblers (64 vs 55 percent, p < 0.001), moderate-risk/problem gamblers (10 vs 6 percent, p < 0.001), to report higher psychological distress (49 vs 43 percent, p < 0.001) and increased time at home during COVID-19 (85 vs 80 percent, p < 0.001), whereas no difference was seen in the reporting of increased alcohol drinking (9 vs 9 percent, p = 0.94). Swedish respondents were more likely to report past-year gambling on online horse race betting (21 vs 3 percent, p < 0.001), land-based horse race betting (11 vs 3 percent, p < 0.001), online sports betting (21 vs 17 percent, p < 0.01), land-based sports betting (12 vs 8 percent, p < 0.001), online poker (5 vas 4 percent, p = 0.03) and online bingo (8 vs 7 percent, p = 0.04), whereas no significant differences were reported for online casino (9 vs 10 percent, p = 0.50), land-based casino (4 vs 4 percent, p = 0.86), or electronic gambling machines (5 vs 6 percent, p = 0.21). Swedish respondents were significantly more likely to report increased gambling during COVID-19 (6 vs 4 percent, p < 0.01).

In the sub-sample of gamblers (n = 1,301 in Sweden, and n = 1,098 in Denmark), Swedish respondents again were more likely to be moderate-risk/problem gamblers (15 vs 10 percent, p < 0.001), to fulfil criteria of psychological distress (49 vs 44 percent, p = 0.03), to report spending more time at home (84 vs 78 percent, p < 0.001), but not increased alcohol drinking (10 vs 9 percent, p = 0.75), and they were no longer significantly more likely than Danish respondents to report increased gambling (9 vs 7 percent, p = 0.10).

## 4 Discussion

The present study aimed to examine self-reported changes in gambling habits during the COVID-19 pandemic in Denmark, and which factors are associated with a changed gambling pattern during COVID-19. The main findings are that a limited minority of respondents reported increased gambling during the pandemic, comparable to the proportion reporting a decrease. People reporting increased gambling hade a more severe gambling pattern, and were more likely to report being more at home during the pandemic, and had a higher degree of mental distress. Importantly, although few individuals reported initiating new gambling types during the pandemic, these individuals were particularly likely to have a more severe gambling pattern. Rates of gambling problems were high in the minority reporting initiating new gambling types during the pandemic. A secondary aim, given the similarity in research design with a study from the neighbor country Sweden, was to compare the changes in gambling patterns in these countries. Here, the main finding was that although Danish respondents were somewhat more likely to be men, a known risk factor of problem gambling, Swedish respondents reported higher rates of moderate-risk/problem gambling, and higher prevalence of increased gambling during COVID-19. In the sub-sample of gamblers specifically, a significant difference in the reporting of increased gambling was no longer seen between the countries.

Altogether, the present study demonstrates that only a minority of the population may have increased their gambling during the pandemic, such that a general increase in gambling behavior cannot be concluded from this self-report survey. However, as in the previous similar studies carried out in Sweden (Håkansson, 2020a; Håkansson and Widinghoff, 2021), the group reporting increased gambling during the pandemic had a very high rate of moderate-risk or problem gambling. While this proportion was not quite as high in Denmark as in the previous Swedish study, it was still demonstrated that almost half of respondents endorsing the item of gambling increase were at least moderate-risk gamblers. Also, for example, in a sample of US land-based casino gamblers, although only a minority increased their online gambling when land-based casinos closed, but the group who moved their gambling to online sources were reported to be a more vulnerable group (Xuereb et al., 2021). The present study, again, confirms the picture that although the vast majority of the population do not change – and particularly do not increase – their gambling, the group which does report an increase present a very high degree of vulnerability.

It should be borne in mind that the changes presented here may in fact reflect a tendency for any person with a gambling problem to have more pronounced changes in gambling behaviors than others; thus, even in individuals reporting a decrease, problem gambling may be high. The present study corroborates this perspective, although gambling severity was higher in ‘increasers’ than in ‘decreasers’, further pointing out that individuals reporting increased gambling during COVID-19 should be assessed for problem gambling and may need to receive attention from treatment providers and preventive initiatives. Thus, any health care facility or social support facility should react to a client who self-reports a perceived increase in gambling during the pandemic. Given the very high rates of people meeting structured screening tool criteria of problem gambling in this group, a further assessment and diagnostic interview may be warranted in these individuals, and referral to structured gambling-oriented addiction treatment should be considered.

As in the first Swedish study (Håkansson, 2020a), in binary analyses, having increased gambling was associated with increased alcohol consumption and increased time at home. In the present study, time at home remained a significant correlate of increased gambling in the adjusted analyses, and alcohol consumption did not, whereas the opposite was seen in the Swedish study from April/May, 2020 (Håkansson, 2020a). The second Swedish study, carried out in November, 2020, demonstrated the same associations of increased gambling as the present study, with both problem gambling, mental distress and increased alcohol consumption (although not with time spent at home, Håkansson and Widinghoff, 2021). It is difficult to conclude that these differences are due to other factors than the statistical model and which variables were included there. Thus, in both settings, it appears that people reporting increased gambling during COVID-19 also have a degree of spending more time at home and influence on alcohol consumption. Again, the present study confirms that life-style changes are likely associated with the subgroup reporting to have changed their gambling behavior during the pandemic.

In the present study in Denmark, gender was not associated with the reporting of increased gambling during COVID-19 in the full study sample. However, within the sample of gamblers (all respondents who did not endorse being a non-gamblers both now and prior to the pandemic), women were significantly more likely to report an increase, and this association only marginally lost its statistical significance in the regression analysis. It has been shown previously that while men are typically over-represented among problem gamblers, female problem gamblers may display a higher degree of severity, and higher rates of psychiatric comorbidity, than male problem gamblers (Håkansson and Widinghoff, 2020a). In a Scandinavian setting, in recent years, it has been shown that in highly online-based gambling markets, women's risk of endorsing problem gambling criteria may be comparable to that of men, in some contrast to what has been seen traditionally (Håkansson and Widinghoff, 2020b). However, while female gender was associated with increased gambling in the present study, in the sub-sample of gamblers, the same was not seen in the corresponding Swedish study used for comparison (Håkansson and Widinghoff, 2021). Thus, it cannot be excluded that women may present a higher risk of increasing their gambling habits as a result of the COVID-19 pandemic, but this clearly requires further study and cannot be concluded from the documentation from the two countries discussed here.

The present study used a methodology very similar to the one used in two previous Swedish studies, i.e. carried out in a neighbor country with a very similar societal structure and welfare system (Håkansson, 2020a; Håkansson and Widinghoff, 2021), and therefore allowed for direct statistical comparisons. In contrast to Denmark, Sweden never applied formal lockdown procedures during COVID-19. Denmark applied lockdown procedures during the spring months of 2020, and thereby during a substantial part of the period of time studied here. Thereafter, new formal lockdown procedures were introduced approximately at the time of the present study, and thereby should not significantly impact the results of the study. However, although the COVID-19 restrictions to society have been more extensive in Denmark than in Sweden (Yarmol-Matusiak et al., 2021), no large differences were seen in the rates of respondents reporting increased or decreased gambling. The percentage of respondents increasing their gambling was somewhat lower in the present Danish study, compared to the later Swedish study, but the difference reached statistical significance only in the full analysis, and not in the sub-sample of gamblers. Likewise, the percentage of respondents reporting decreased gambling was comparable to the corresponding figure in the second Swedish study. Altogether, despite different COVID-19 approach with respect to formal lockdown, substantial differences in self-reported gambling changes could not be seen. In both settings, high rates of online gambling are reported, and the Swedish data included a somewhat larger number of gamblers and a larger number of individuals with at least moderate-risk gambling. Although gambling opportunities were reduced during the early months of the pandemic, even for sports bettors online (due to the lockdown of sports worldwide), online gamblers generally have not seen their gambling opportunities substantially altered during the pandemic. The large role of online gambling in these settings may explain the relatively limited changes in gambling patterns seen during the pandemic.

Beyond the specific issue of gambling and COVID-19, the Sweden-Denmark comparison demonstrated somewhat higher rates of estimated mental health problems, and a higher percentage who reported spending more time at home, in Sweden than in Denmark. This may seem to be in some contrast to differences in COVID-19 restrictions, including the absence of formal lock-down procedures in Sweden (Yarmol-Matusiak et al., 2021). These differences were seen both in the full sample and in the sub-sample of gamblers. Both these findings are difficult to fully explain from the present type of data. However, a Danish two-wave study demonstrated that from March to April, 2020, a decreasing viral transmission and a certain relief of restrictions were associated with a significant improvement in mental health in the Danish population (Sönderskov et al., 2020b). As the present study, and the contrasting study from Sweden, were carried out in the late part of 2020, it is at least theoretically possible that the continued trends in virus transmission and high mortality may have affected mental health in the Swedish population. In Sweden, mortality rates remained substantially higher than in Denmark through 2020 (Yarmol-Matusiak et al., 2021), which may potentially have an impact on perceived mental health. It also cannot be excluded that the perceptions of this prolonged crisis may have affected the high perceived impression of time spent at home in Sweden, although no data are available to confirm this.

Early during the pandemic, concerns were expressed about the risk of mental distress due to COVID-19, and that such reports in the psychiatric clinical setting were reported to follow the decision of lock-down of society in Denmark (Rohde et al., 2020). The present study demonstrates that people with poor mental health were at particular risk of reporting increased gambling habits. It shall be borne in mind that this finding does not imply a causal relationship between these factors. However, this finding may have the implication that treatment services and prevention and screening efforts should address potentially increasing gambling patterns among the health hazards potentially seen in patients with poor mental health, particularly during the pandemic. Likewise, the link between spending more time at home, and self-reported increase in gambling, needs to be addressed further. Social isolation, stay-at-home orders, home schooling and advice to work at home, are altogether factors which may increase gambling opportunities. Gambling, among other mental health issues, should be addressed in individuals with clearly increasing time spent at home.

The present study has a number of limitations. First, it assessed self-reported subjective data, and does not have access to actual gambling data. In self-report data collections, recall bias is a well-known challenge, both in other addictive behaviors (Gmel and Daeppen, 2007) and in the field of gambling (Dussault et al., 2019). Also, the study is based on a web survey inviting people involved in a web panel of a market survey company, and therefore, it may reach individuals with a higher degree of online use and a higher degree of interest in online gambling than in the rest of the population. Thus, given the online collection of data, study findings cannot be generalized to any country in the world, such as countries with a more land-based gambling market. However, the study of associations within the study sample, as well as the comparison to one different country and with the same study methodology, can still provide important insights for the present type of gambling market. In addition, due to the need to keep an online survey brief, detailed data about gambling patterns, such as gambling frequency or money spent gambling, were not included in the study. Further, the study did not use any longitudinal measure of gambling, and should be repeated in the future, particularly in later phases of the pandemic.

## 5 Conclusion

In conclusion, gambling patterns were reported to have increased during COVID-19 in only a minority of the Danish population, but mental distress and more problematic gambling severity were associated with the reporting of increased gambling. Thus, despite limited overall changes in gambling habits during COVID-19, people increasing their gambling during the pandemic may have particular risk. In comparison to a setting without formal lockdown procedures during COVID-19 (Sweden), changes in gambling habits were similar, and the increase in gambling even rather tended to be larger in Sweden. Settings with a high degree of online gambling may see a different picture of gambling habits during COVID-19 compared to less online-based gambling markets.

## Declarations

### Author contribution statement

Anders Håkansson: Conceived and designed the experiments; Performed the experiments; Analyzed and interpreted the data; Contributed reagents, materials, analysis tools or data; Wrote the paper.

### Funding statement

This work was supported by the AB Svenska Spel and the Region Skåne health care organization.

### Data availability statement

Data will be made available on request.

### Declaration interests statement

The authors declare no conflict of interest.

### Additional information

No additional information is available for this paper.
